# Prediction of IGS RTS Orbit Correction Using LSTM Network at the Time of IOD Change

**DOI:** 10.3390/s22239421

**Published:** 2022-12-02

**Authors:** Beomsoo Kim, Jeongrae Kim

**Affiliations:** School of Aerospace and Mechanical Engineering, Korea Aerospace University, Goyang-si 10540, Gyeonggi-do, Republic of Korea

**Keywords:** GNSS, RTS correction, RTS prediction, PPP, LSTM

## Abstract

The international GNSS service (IGS) real-time service (RTS) provides orbit and clock corrections for the global navigation satellite system (GNSS) via the internet. RTS is widely used for real-time, precise positioning and its data is transmitted via the internet. Intermittent data loss can occur and cause positioning accuracy degradation. RTS data has a discontinuity when the issue of data (IOD) changes every two hours. If the signal loss occurs immediately after the IOD change, then the performance of the RTS prediction degrades significantly. We propose an adjustment method to make the RTS data across the IOD change, which makes it possible to use long RTS data for building a prediction model. The proposed adjustment method is combined with a long-short-term memory (LSTM) network to improve long-period prediction accuracy. Experiments with GPS and RTS were performed to evaluate the RTS orbit prediction accuracy. The LSTM with the IOD adjustment outperforms other polynomial prediction methods, and the positioning accuracy with the predicted RTS orbit correction shows a significant improvement.

## 1. Introduction

While precise satellite orbit and clock products are necessary to perform the precise point positioning (PPP) technique, any errors that exist in the global navigation satellite system (GNSS) absolute positioning must also be modeled. The international GNSS Service (IGS) real-time service (RTS) correction or IGS ultra-rapid product is mainly used to correct the orbit and clock error components of navigation satellites for real-time applications. However, the IGS ultra-rapid product makes use of predicted information, and the error of the clock increases over time and does not meet the requirements of PPP [1,2].

The RTS correction provides information about the navigation satellite orbit and clock correction over the internet, and the user can obtain accurate orbit and clock information by applying the RTS correction to the navigation satellite orbit and clock from the navigation message. However, RTS availability is not 100% due to issues such as caster failure or user’s internet connection, and it may cause a problem with applying RTS orbit and clock corrections in real-time. If RTS correction information is not available during the real-time PPP, the positioning error may increase rapidly. Therefore, it is necessary to predict the RTS correction during the signal interruption period to prevent the error from increasing.

Several studies have been carried out to predict the RTS correction. First, the prediction has been proposed using a polynomial model in a short time interval [3]. Some studies have performed clock correction prediction by modeling the RTS correction and IGS ultra-rapid product as a harmonic function [4,5,6], and another study applied the Fourier series to the prediction [7]. Studies have also been proposed that apply machine learning to prediction. For example, there was a case where RTS prediction was performed by combining an auto-regressive moving average (ARMA) with a neural network or genetic algorithm [8,9] and a case where RTS correction was predicted with a long-short term memory (LSTM) [10,11]. In general, these advanced algorithms show better performance over a long period interval than the polynomial method.

Information about the issue of data (IOD) is included in the navigation message transmitted by GNSS and RTS. IOD means the issue number for the transmitted message. IOD includes IOD ephemeris (IODE), which is an IOD value for the orbit, and IOD Clock (IODC), which is a value for the clock. The IOD changes if the navigation message is changed every 2 h. The RTS correction is not an absolute value, but an additional value to the broadcast ephemeris and clock in the navigation message. When the broadcast ephemeris is changed every two hours, the RTS correction changes according to the new broadcast ephemeris. When the IOD changes, both the RTS correction and the satellite orbit calculated from the navigation message change discontinuously. A special situation is when the RTS signal outage or loss occurs immediately after the IOD change. In this situation, it becomes very difficult to predict the RTS correction. This is because the prediction method does not have enough fitting data for the prediction. For example, if the IOD changes at 2:00 P.M. and the RTS signal loss occurs at 2:03 P.M., we have only three minutes of fitting data for the RTS prediction process. To solve this problem, the authors proposed using the orbit change value to eliminate the RTS correction discontinuity at the IOD change. Although this special situation is rare, e.g., a few minutes during two-hour constant broadcast ephemeris transmission (between consecutive IOD changes), it is important for PPP that requires continuous orbit and clock corrections to resolve ambiguities. The usefulness of the concept was demonstrated using a polynomial prediction model at the time of IOD change [12]. The polynomial model uses shorter fitting data than other extended prediction models, e.g., LSTM, and the accuracy improvement by the IOD change adjustment may differ from the extended models.

In this study, we propose a prediction method for RTS orbit correction using the LSTM network by removing the discontinuity of RTS correction when signal interruption occurs after the IOD change. Since RTS clock corrections have white noise characteristics [3], the performance improvement from the LSTM was not significant as the amount of computation for the RTS clock correction increased; so, only the RTS orbit correction was predicted with the LSTM network. By applying the orbit difference between orbits before and after the IOD change to RTS correction, discontinuities were removed and used for LSTM network updates. Compared to the polynomial prediction method, the LSTM needs more observation data to update its trained network. After the IOD change, there is very little data available for the update and the LSTM performance decreases significantly. The performance of the proposed method was verified by comparing the RTS orbit prediction and the PPP results with the existing prediction methods. Because there was no significant performance improvement in the prediction of the clock correction using the LSTM network, the 0th-order polynomial was used in the clock correction prediction in all cases.

## 2. Background and Methods

### 2.1. Overview of RTS Correction

RTS correction is streamed over the internet in the radio technical commission for maritime services (RTCM) state space representation (SSR) format. The internet protocol uses networked transport of RTCM via internet protocol (NTRIP) [13,14,15,16].

**Figure 1:**
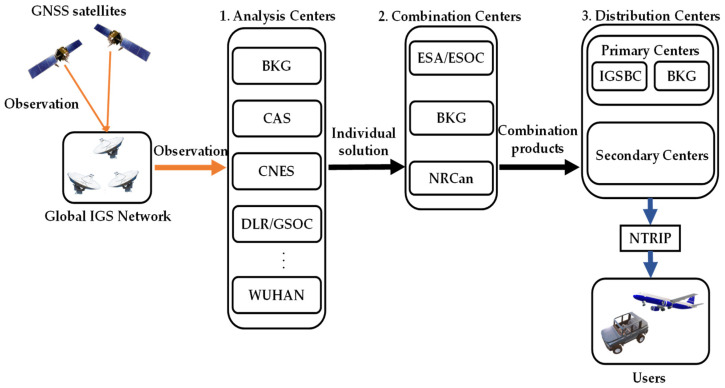
Flowchart for generating RTS orbit and clock corrections.

RTS corrections are produced by the IGS real-time workgroup (RTWG), as shown in Figure 1 [15,17]. The first step is for real-time analysis centers (RTAC), including BKG, CAS, CNES, DLR, ESA, GFZ, GMV, NRCan, SHAO, UPC, and WUHAN, to produce individual solutions using observations from IGS GNSS network. The second step is for the combination centers, BKG, and NRCan, to combine the products and produce individual solutions. Realignment, detection, and elimination of outliers, and averaging are performed during this process. The final step is for distribution centers to stream products over the internet using the NTRIP client. IGS Central Bureau (IGSCB) and BKG are the two primary product distribution centers, and there are also several secondary distribution centers. 

IGS RTS provides several products for orbit and clock correction, such as Table 1 [15]. The SSRA01IGS1 and SSRC01IGS1 use a single-epoch combination to generate a 5-s interval solution, while the rest use a Kalman filter to generate a 60-s orbit and 10-s interval clock solution. The origin of the coordinate system is also divided into center of mass (CoM) and antenna phase center (APC) according to the type of RTS product [18]. The IOD of the RTS correction is included in the orbit information, and the correction must be applied to the navigation message corresponding to the IOD of the RTS correction. Detailed information on IGS RTS is available online [15].

Since RTS orbit correction is provided in radial, along-track, and cross-track (RAC) coordinate systems, transformation to the Earth-centered Earth-fixed (ECEF) coordinate system is essential for applying to navigation message via the transformation equation as

(1) $${\hat{e}}_{r}=\frac{\vec{r}}{\left|\vec{r}\right|}\;\;\;\;\;\;\;\;\;\;\;\;\;{\hat{e}}_{c}=\frac{\vec{r}\times \dot{\vec{r}}}{\left|\vec{r}\times \dot{\vec{r}}\right|}\;\;\;\;\;\;\;\;\;\;{\hat{e}}_{a}={\hat{e}}_{c}\times {\hat{e}}_{r}$$

(2) $${\vec{RTS}}_{ECEF}=\left[{\hat{e}}_{r}\;\;\;{\hat{e}}_{a}\;\;\;{\hat{e}}_{c}\right]\;{\vec{RTS}}_{RAC}$$

where $\vec{r}$, $\dot{\vec{r}}$ are position and velocity vectors of the satellite. ${\hat{e}}_{r}$, ${\hat{e}}_{a}$, and ${\hat{e}}_{c}$ are unit vectors of RAC direction. ${\vec{RTS}}_{RAC}$ is the orbit correction vector in the RAC coordinate system and ${\vec{RTS}}_{ECEF}$ is the orbit correction vector in the ECEF coordinate system.

**Figure 2:**
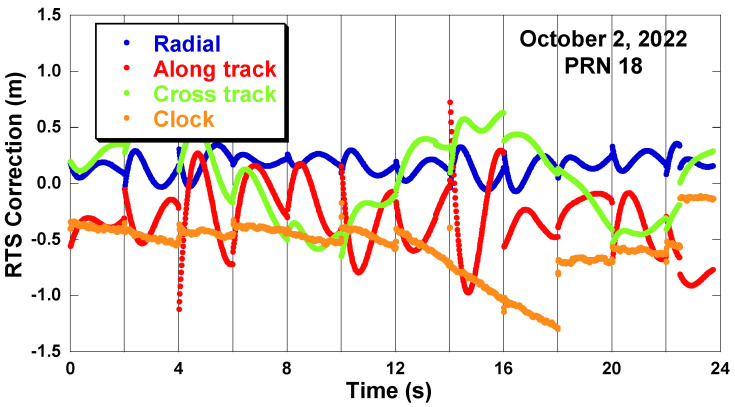
RTS correction discontinuity at IOD changes (GPS PRN18, 2 October 2022).

Figure 2 is data of the GPS pseudo-random number (PRN) 18 satellite, showing that the orbit correction changes discontinuously when the IOD is updated every 2 h. Even the variation pattern of the RTS signals has completely changed after the IOD is changed. Therefore, it is not possible to use the old RTS correction after the IOD change. The behavior of the clock correction changes is slightly different from the orbit correction changes. The magnitude of the clock correction change is relatively smaller than that of the orbit correction change. At some IOD changes, there are no clock correction jumps [19]. Therefore, it is difficult to predict the RTS correction when the signal interruption occurs.

### 2.2. Analysis of the RTS Correction at the Time of IOD Change

To analyze the changes in the RTS corrections at the time of the IOD change, the mean change of the RTS corrections at each IOD change was calculated. SSRA03IGS1 (IGS03) data streamed from 28 September to 2 October 2022, was analyzed. At 12 IOD changes per day, a total of 58 changes were considered, excluding the start and end of the data.

**Figure 3:**
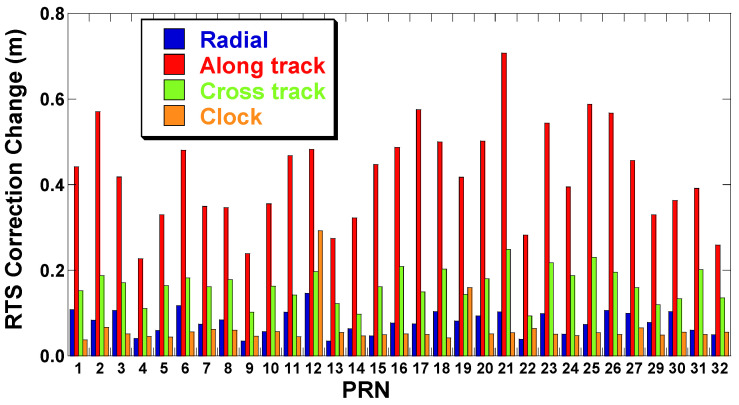
The magnitude of the RTS correction differences at IOD changes (GPS satellites, 28 September–2 October 2022).

Figure 3 is obtained by averaging the magnitude of the discontinuities that occurred in the GPS orbit and clock correction at the time of the IOD change. The magnitude of the change varies with the direction of the orbit correction. The change in the along-track direction was the greatest, with an average of 0.42 m, while the change in the cross-track direction was the smallest, with an average of 0.08 m. The reason why change is the greatest in the along-track direction is that the change in orbit of the satellite is the greatest in the velocity direction. When the 3D orbit change is calculated, the mean is 0.46 m, and the clock correction is 0.06 m, which is similar to the radial direction.

**Figure 4:**
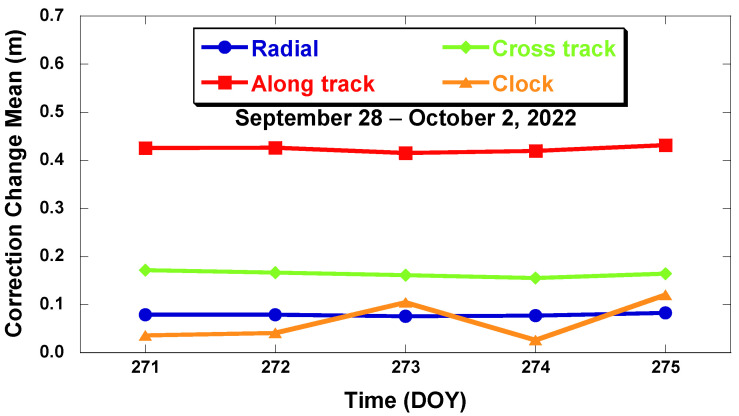
Daily magnitude of the RTS correction difference at IOD changes (GPS satellites, 28 September–2 October 2022).

Figure 4 shows the result of the mean change in RTS correction per day. It was calculated by averaging the magnitude of the change of all GPS satellite corrections by date. As shown in Figure 4, the along-track direction has the greatest magnitude of change with a mean value of 0.46 m.

### 2.3. Change in RTS Correction and Navigation Message at the Time of IOD Change

When the IOD changes, a discontinuity occurs in the orbit generated from the navigation message and the RTS correction. When predicting RTS correction, it is difficult to predict with discontinuous data, so prediction should only be performed with the data received after the IOD change. However, due to the small amount of received data, a large prediction error occurs. If the difference in orbit calculated by the navigation message before and after the IOD change is applied to the RTS correction using this method, then it is possible to predict the RTS correction after the IOD change and the RTS correction becomes continuous.

**Figure 5:**
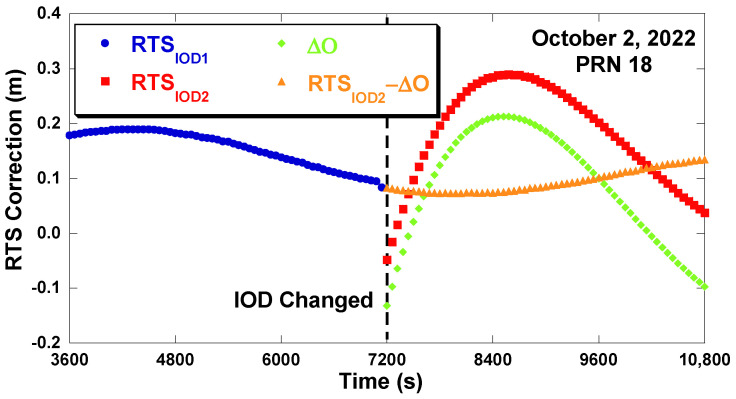
RTS orbit correction and difference at the time of the IOD change (28 September 2022).

Figure 5 shows the change in RTS radial correction and the change in the orbit of the navigation message before and after the IOD change. $RTS_{IOD1}$ and $RTS_{IOD2}$ are the RTS corrections before and after the IOD change, respectively. After the IOD change, RTS has a change of 0.13 m, and its variation behavior becomes completely changed. The satellite orbit difference $\Delta \vec{O}$ is calculated by subtracting the derived orbits of the navigation message before and after the IOD change as follows:

(3) $$\Delta \vec{O}={\vec{O}}_{IOD2}-{\vec{O}}_{IOD1}$$

where ${\vec{O}}_{IOD2}$ is the orbit of the satellite calculated by the navigation message after the IOD change and ${\vec{O}}_{IOD1}$ is the orbit calculated by the navigation message before the IOD change. If $\Delta \vec{O}$ is applied to the correction received after the IOD change, it is possible to obtain the continuously changing correction from the previous IOD correction as

(4) $$\Delta {\vec{RTS}}_{IOD2-IOD1}={\vec{RTS}}_{IOD2}-\Delta \vec{O}$$

$\Delta {\vec{RTS}}_{IOD2-IOD1}$ is the RTS correction by removing the discontinuity that occurred at the IOD change. Therefore, it is possible to update the LSTM network with all data regardless of the change in IOD, even if a signal interruption occurs after the IOD change.

### 2.4. LSTM Network

When learning data over a long period of time with a recurrent neural network (RNN), the weights are not updated correctly due to the gradient loss that occurs during the back-propagation process [20]. To solve this long-term dependency problem of RNNs, the LSTM technique was introduced [21].

**Figure 6:**
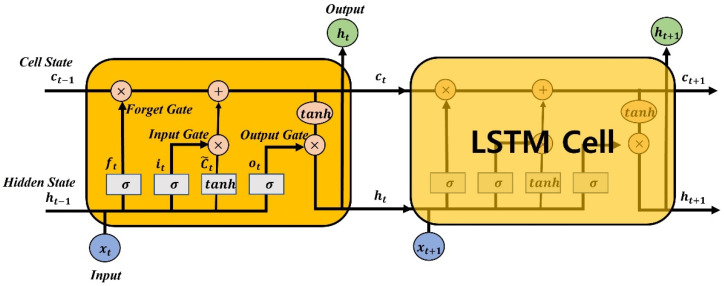
LSTM cell structure.

As shown in Figure 6, the LSTM cell has the input $x_{t}$ and the output $h_{t}$, which is also known as the hidden state [22]. There are also three gates (forget, input, and output) that allow long-term data to process. Each gate computation can be considered as a fully connected layer [23]. The output of each gate, $f_{t}$, $i_{t}$, and $o_{t}$, can be computed as

(5) $$f_{t},\;i_{t},\;or\;o_{t}=\sigma \left(W_{x}x_{t}+W_{h}h_{t-1}+b_{i}\right)$$

where σ(x), known as the Sigmoid activation function, can map a real value to the interval from 0 to 1 to describe how much information passes through. An output value of 1 indicates that no data is passed, and an output value of 0 does that all data is passed. $W_{x}$ is the weight for the current input $x_{t}$ and $W_{h}$ is the weight for the output of the previous LSTM cell $h_{t-1}$. And $b_{i}$ represents the bias for each gate. In Figure 6, the cell state passes through the entire cell in the LSTM layer and acts as a memory. The current cell state $C_{t}$ can be calculated using input and forget gates as

(6) $${\tilde{C}}_{t}=tanh\left(W_{xc}x_{t}+W_{hc}h_{t-1}+b_{c}\right)$$

(7) $$C_{t}=f_{t}C_{t-1}+i_{t}{\tilde{C}}_{t}$$

where ${\tilde{C}}_{t}$ is the input cell state and is calculated as a fully connected layer using the hyperbolic tangent function tanh(x). The input gate determines how much of the current input $x_{t}$ should be updated to $C_{t-1}$. The forget gate determines which data is saved or forgotten and prevents gradient loss problems. Due to its gated structure, especially the forget gate, LSTM is an effective and scalable model for many long-period sequential learning problems [24]. Finally, the output of the current LSTM cell can be calculated as

(8) $$h_{t}=o_{t}tanh\left(C_{t}\right)$$

Using the output gate, we can only export the part we want to send as output.

**Figure 7:**
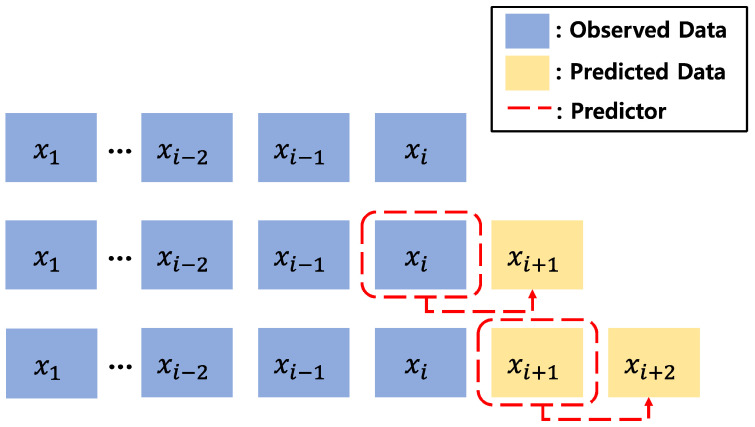
One-step forward LSTM network prediction method.

The LSTM network implemented in this study is trained to predict the future with one-step forward. Figure 7 depicts the prediction method of the one-step forward LSTM network. This method consists of LSTM network update and prediction. LSTM network update is performed at the point of each data observation, and one-step forward data is recursively predicted in the prediction stage.

## 3. Data Processing

The LSTM prediction experiments were performed on the assumption that the RTS correction outage occurs immediately after the IOD change. The RTS was assumed to be interrupted for 3000 s from 180 s after the IOD change, and since IOD changes every 2 h, a total of 12 data loss periods per day was assumed. The prediction performance of the LSTM network was compared with the polynomial model.

To predict RTS orbit and clock correction using a polynomial model, it is necessary to select the best polynomial order for the prediction. In this study, the order was chosen based on references [3,25]. RTS orbit correction can be approximated by the 2nd to 4th-order polynomial model, and clock correction uses 0 or 1st-order [3]. Based on the results of Hadas’s research [3,25], the 2nd-order showed good results for the prediction of orbit correction up to 5 min, and the 3rd or higher order showed good results for long-term predictions. When a 4th-order polynomial was used for a 2-h fitting period, the standard deviation (STD) of the fitting error was 4 times smaller than that of the 3rd order. Since the RTS clock correction has no great tendency to follow 1st or higher-order polynomials, a 0th-order (constant) polynomial can be used [3]. In this study, two polynomial models were used to predict the RTS orbit correction. When the fitting data was sufficient by using the data before and after the IOD change, the 4th order was used. And when the fitting data was insufficient by using the data only after the IOD change, 1st order was used.

SSRA03IGS1 data for a total of 4 weeks from 7 July to 3 August 2022, was used to train the LSTM network for the orbit correction prediction. For the pre-processing of LSTM network training data, all data was separated and standardized for each IOD interval. The number of LSTM cells is 50 and they have been trained to predict future data one-step ahead using the adaptive moment estimation (Adam) optimizer. Based on first-order gradients, the Adam optimizer adaptively calculates the learning rate for each weight and is one of the most efficient stochastic optimization algorithms [26]. Adam is a method that combines the advantages of adaptive gradient (AdaGrad) and root mean square propagation (RMSProp) [26]. Since the LSTM network is trained with data before the prediction test is preformed, the LSTM network update is performed in real-time using the RTS orbit correction received before the interruption. 

Two types of LSTM experiments were performed with different lengths of update data. One updated the LSTM network at a short data interval (180 s) after the IOD change. The other updated the LSTM network with a long data interval (3600 s) before and after the IOD change. The latter utilized the concept of the orbit difference adjustment after the IOD change. The polynomial experiments have the two types as well—one with a short length of data for polynomial fitting and the other with a long length of data.

When considering the IOD change, network update and 4th order polynomial fitting were performed with 3600 s of data before and after the IOD change. When ignoring the IOD change, the network update and 1st order polynomial fitting were performed with 180 s data right after the IOD change. With the short 180 s fitting data, 4th order polynomial fitting is not suitable and 1st order was used instead. After the update or fitting, the RTS prediction was executed for 3000 s. All four cases are summarized in Table 2. As mentioned earlier, LSTM was not used for the RTS clock correction, and all four cases used the same 0th order polynomial for the clock prediction.

**Figure 8:**
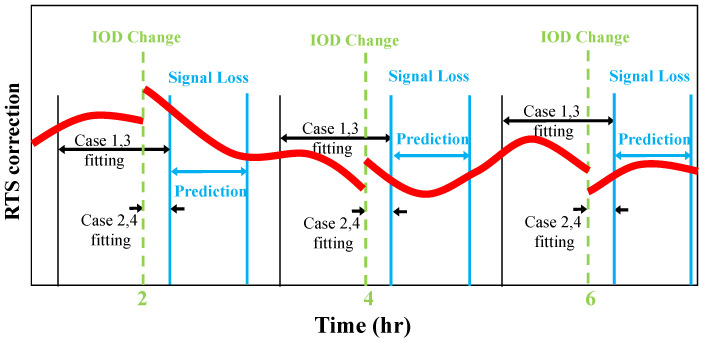
Time intervals of the LSTM and polynomial update and prediction.

Figure 8 depicts the time intervals of the update and prediction. The test was performed a total of 12 times per day, at every two-hour IOD change interval. An RTS interruption occurred 180 s after the IOD change, and the prediction of RTS correction was performed for 3000 s. Cases 1 and 3 used 3600 s of data before and after the IOD change, while Cases 2 and 4 used only 180 s of data after the IOD change.

## 4. RTS Prediction Results

Prediction of the RTS corrections was performed using the four methods, cases 1 through 4. Five days’ worth of data were processed to evaluate the performance of the LSTM prediction method with the consideration of the IOD change.

**Figure 9:**
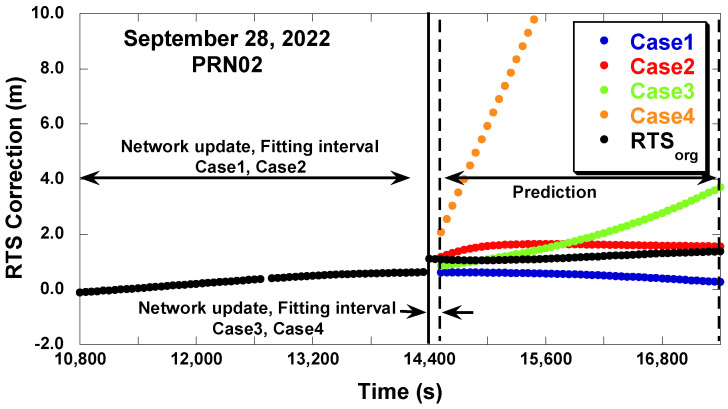
RTS correction prediction results (GPS PRN02 along track, 28 September 2022).

Figure 9 shows the original RTS correction and the prediction results in the along-track direction. With the adjustment of the IOD change differences—Cases 1 and 3—the predicted correction is linked to the raw RTS data before the IOD change (~14,400 s). Especially Case 1 (LSTM) shows continuous RTS data from 10,800 s to 17,000 s. Without the adjustment of the IOD change difference, seen in Cases 2 and 4, the predicted correction is linked to the raw RTS data after the IOD change (~145,800 s) and has a jump from the raw RTS data before the IOD change. Case 4 diverges in the beginning and it proves that the short fitting interval is not especially suitable for the polynomial prediction.

**Figure 10:**
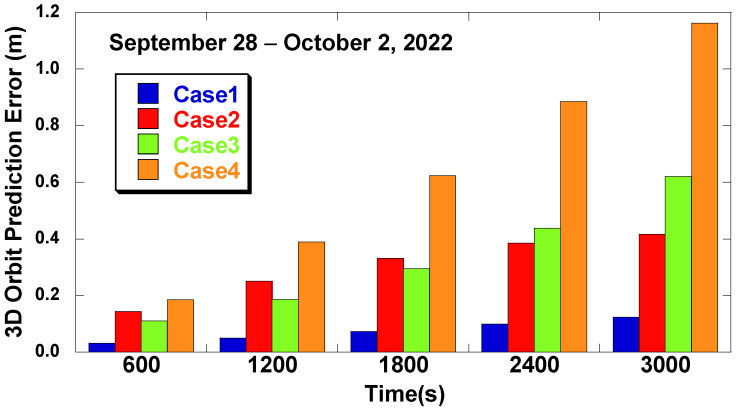
RTS orbit correction error at different prediction intervals (28 September–2 October 2022).

The accuracy of the predicted corrections was calculated by adding the predicted results to the orbit derived by the navigation message and by comparing them to the IGS precise ephemeris. Figure 10 shows the mean of the orbit prediction errors for each prediction interval. Label 1800 s represents the prediction error up to 1800 s after the RTS signal loss. In the short prediction intervals, e.g., 600 s, the error difference between the cases is not significant, but the difference grows with the prediction intervals. Case 1 outperforms Case 2, and the error is reduced by 71.8% for the 3000 s interval. It proves the effectiveness of the IOD change adjustment. The error reduction of Case 1 over Case 3 is 80.6% for the 3000 s interval. It proves the effectiveness of the LSTM network over the polynomial method for a long prediction interval.

## 5. PPP Performance Analysis Using RTS Predictions

The positioning accuracy with the predicted RTS corrections was evaluated. For the real-time test, only GPS data was used, and the prediction results were applied to the GPS broadcast ephemeris. PPP was performed by RTKLIB, a real-time precise positioning program. RTKLIB is an open-source program developed by Takasu of the University of Tokyo and is now widely used to run PPP [27]. The functionality of real-time prediction of the RTS correction using the LSTM and polynomial methods was implemented in RTKLIB, and the real-time positioning accuracy was evaluated. The GPS observation data from the IGS ONS1 station in Sweden was used via NTRIP, and the same RTS prediction data of the previous section was applied to RTKLIB. GPS broadcast ephemeris and clock data from NTRIP were used. RTS signal loss was simulated by blocking the RTS data stream from the internet to the RTKLIB PPP software. The experiments were performed every 2 h for 5 days from 28 September to 2 October 2022, and the true position of the ONS1 station was obtained from the IGS SINEX file. Case 4 was excluded from the PPP experiments because the PPP results diverged from the start due to a large prediction error. A total of four PPP experiments were performed simultaneously using Cases 1–3 and the original RTS corrections.

**Figure 11:**
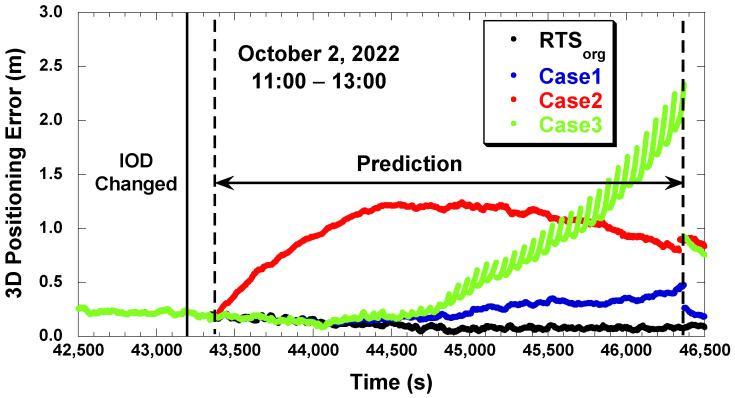
PPP positioning error with different prediction methods (2 October 2022).

Figure 11 shows the PPP positioning error with the predicted RTS data from 11:00 to 13:00 UTC on 2 October 2022. The IOD change occurred at 12:00 (43,200 s) and the prediction lasted for 3000 s, from 43,380 s to 46,380 s. ”${\mathrm{RTS}}_{\mathrm{org}}$” is a PPP result of using original uninterrupted RTS corrections without blocking the RTS stream and is presented for reference. The PPP results with the predicted RTS correction, Cases 1 to 4 might not be equal or better than the PPP results with the original RTS correction, RTS_org, but they were close to the RTS_org with a better prediction. With the adjustment of the IOD change difference, in Cases 1 and 3, the positioning error remained low at the beginning of the prediction interval. Without the adjustment of the IOD change difference, as seen in Case 2, the positioning error grew rapidly at the beginning of the prediction interval. The IOD change adjustment was effective in both positioning and correction prediction. Case 3 had a similar positioning accuracy to Case 1 up to 1000 s of the prediction interval, the 44,380 s mark in Figure 11, but Case 3’s positioning error rapidly grew after that point. This behavior, the advantage of the LSTM method over the polynomial method for a long prediction interval, was consistent with the RTS prediction results in Figure 10. After the prediction was over, the error of Case 1 quickly converged to the original accuracy, but the error of other cases required extra convergence time due to their large positioning error during the prediction interval.

**Figure 12:**
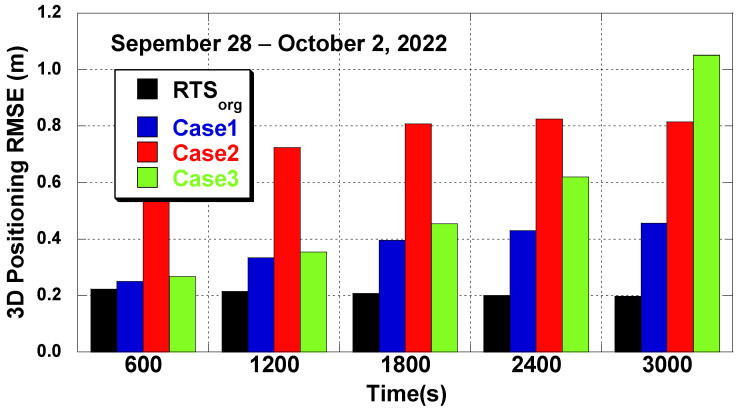
Positioning error at different prediction intervals (ONS1, 28 September–2 October 2022).

Figure 12 compares the PPP positioning error RMSs from different prediction intervals. The five-day positioning errors are averaged. As the RTS correction prediction results show in Figure 10, Case 1 shows the best PPP accuracy. The growth of the positioning error of the LSTM network in Cases 1 and 3 was modest along with the growth of the prediction interval, but the growth of the positioning error of the 4th order polynomial, seen in Case 2, was rapid with the growth of the prediction interval. The positioning error reduction by Case 1 was 36.5% over Case 2 and 54.2% over Case 3. The error reduction ratio was slightly less than the RTS prediction error reduction ratio, 71.8% and 80.6%. This is because the same prediction method, constant value prediction, was used for the clock prediction even with the LSTM methods. The positioning error also included the error contribution from the clock prediction error. In addition, the STD of all 3D positioning errors was 0.06 m, 0.16 m, 0.21 m, and 0.59 m for ${\mathrm{RTS}}_{\mathrm{org}}$, in Case 1, Case 2, and Case 3, respectively. The error STD reduction ratio was similar to the error RMS reduction ratio.

Table 3 shows the statistics of horizontal and vertical positioning errors over 5 days, and similar to other results, the statistics covered the error during the prediction interval. Compared with the case where no RTS interruption occurred, the error mean of the proposed method, Case 1, increased by 0.15 m and 0.17 m in the horizontal and vertical directions, respectively. Compared to the polynomial method in Case 3, the error reduction by Case 1 was greater in the vertical direction, both in the mean and the STD. The mean error reductions by Case 1 over Case 3 were 37.0% (horizontal) and 51.8% (vertical). The STD of the error reductions by Case 1 over Case 3 were 63.7% and 70.2%. The reduction in STD was slightly greater than the reduction in mean. 

## 6. Conclusions

The LSTM network is applied to predict IGS RTS orbit correction during periods of data loss. The LSTM method offers better accuracy than the polynomial method when the prediction period is long. IGS RTS corrections are generated to adjust the orbit and clock information of the GNSS navigation message, which is regularly updated, and the updates are identified by the IOD number. When the IOD changes, the corresponding RTS correction changes abruptly and the RTS correction is no longer continuous after the IOD change. This discontinuity makes it difficult to predict the RTS correction if the signal loss occurs immediately after the IOD change. We propose an offset value to minimize the RTS discontinuity by subtracting the navigation message orbits before and after the IOD changes.

The proposed method was evaluated with five days of GPS and RTS orbit correction data. The evaluation focused on the prediction performance when the RTS signal loss occurred immediately after the IOD change. The results of the LSTM network were compared with those of the polynomial method, and the results with the IOD change adjustment were compared with those without IOD adjustments. The prediction interval, corresponding to the signal loss duration, was set to 3000 s. Since the prediction accuracy of the LSTM network for the RTS clock correction was not significant, the constant (0th order polynomial) prediction method was used for both the LSTM and the polynomial methods. 

The LSTM network method with the IOD change adjustment outperforms other methods. The reduction of the prediction error by the LSTM method with the IOD adjustment over the 4th order polynomial method with the same IOD change adjustment is 80.6%. The PPP positioning accuracy with the predicted RTS corrections shows similar behavior as the RTS prediction. The LSTM method with the IOD adjustment outperforms other methods; the vertical position error mean is reduced by 51.8% from the polynomial method without the IOD adjustment and by 47.1% from the LSTM method with the IOD adjustment.

## Figures and Tables

**Table 1:** List of RTS orbit and clock correction products.

Product	Description	Ref Point	Satellite System
SSRA01IGS1	Single-Epoch Combination	APC	GPS
SSRC01IGS1	Single-Epoch Combination	CoM	GPS
SSRA02IGS1	Kalman Filter Combination	APC	GPS, GLONASS, Galileo
SSRC02IGS1	Kalman Filter Combination	CoM	GPS, GLONASS, Galileo
SSRA03IGS1	Kalman Filter Combination	APC	GPS, GLONASS, Galileo, BeiDou
SSRC03IGS1	Kalman Filter Combination	CoM	GPS, GLONASS, Galileo, BeiDou

**Table 2:** List of RTS prediction methods in case of RTS signal loss.

Parameter	Case 1	Case 2	Case 3	Case 4
IOD use	Past + Recent	Recent	Past + Recent	Recent
Prediction Method	LSTM	LSTM	4th Polynomial	1st Polynomial
Fitting length	3600 s	180 s	3600 s	180 s

**Table 3:** Statistics of horizontal and vertical positioning errors (ONS1, 28 September–2 October 2022 unit: meters).

	${\mathrm{RTS}}_{\mathrm{org}}$	Case 1	Case 2	Case 3
H Mean	0.14	0.29	0.42	0.46
V Mean	0.10	0.27	0.51	0.56
H STD	0.04	0.12	0.13	0.33
V STD	0.06	0.14	0.21	0.47

## Data Availability

Not applicable.
